# Positive Education for Young Children: Effects of a Positive Psychology Intervention for Preschool Children on Subjective Well Being and Learning Behaviors

**DOI:** 10.3389/fpsyg.2017.01866

**Published:** 2017-10-26

**Authors:** Anat Shoshani, Michelle Slone

**Affiliations:** ^1^Baruch Ivcher School of Psychology, Interdisciplinary Center Herzliya, Herzliya, Israel; ^2^School of Psychological Sciences, Tel Aviv University, Tel Aviv, Israel

**Keywords:** preschool, intervention, positive-psychology, positive education, mental health, well-being, children

## Abstract

Despite the flourishing in recent years in applications of positive psychology in the field of education, there is a paucity of research investigating positive psychology interventions for preschool children. The present study examined the effects of a positive psychology-based intervention conducted in Israel on children’s subjective well-being, mental health and learning behaviors. Twelve preschool classrooms of 3–6.5 year-olds were randomly assigned to a positive psychology intervention condition or a wait-list control condition. In the intervention condition, during one school year, 160 children experienced eight modules of basic concepts in positive psychology that were adapted to the developmental characteristics of young children and were compared to 155 children in demographically similar control classrooms. Children were administered a pre-test and post-test of subjective well-being measures. In addition, children’s mental health and emotional well-being were measured by parental questionnaires. Preschool teachers completed questionnaires concerning children’s learning behaviors. The findings showed significant increases in subjective well-being and positive learning behaviors among the intervention participants, with no significant changes in the control group. The results highlight the potential of positive psychology interventions for increasing subjective well-being and a positive approach to learning at young ages.

## Introduction

Recognition of the importance of social and emotional development in young children has become a primary priority of early childhood education. Successful negotiation of this developmental period includes, among other acquisitions, the ability to form positive relationships, to establish positive self-esteem, to effectively express feelings and regulate emotions, to persevere and engage positively with challenging tasks, and to adopt a positive outlook in a dynamic environment (Bowman et al., 2000; Shonkoff and Phillips, 2000; Duckworth et al., 2007; Oades et al., 2011). Therefore, the increasing psychological and mental health focus on the foundational early childhood developmental period has led to international interest in promoting socio-emotional development and personal strengths in early childhood education (Honig, 2002).

Many of the competencies acquired during this developmental period are the foundational constructs embraced in the Positive Psychology approach to education that focuses on the optimal functioning of educators and children in the different educational settings. This area of inquiry has flourished recently in the form of Positive Education that seeks to integrate positive psychology elements with educational practices (Seligman et al., 2009; Sin and Lyubomirsky, 2009; Stiglbauer et al., 2013; Shoshani and Steinmetz, 2014). Increasingly, educational intervention programs include positive psychology constructs such as character strengths, gratitude, positive emotions and engagement to improve children’s well-being and mental health. Most of these interventions have been instituted in schools with a paucity of positive psychology interventions for young children in preschool educational settings. Informed knowledge of the effectiveness of these interventions in promoting preschool children’s well-being is sorely lacking. The present study describes the construction of a positive education program applied by trained kindergarten teachers and investigates the efficacy of this program on promoting preschool children’s well-being, strengths and socio-emotional abilities and reducing difficulties.

### The Need for Positive Education in the Preschool

Extensive research has examined children’s developmental needs in preschool settings from the vantage point of school readiness (Pianta et al., 2007). The accumulated data regarding preparedness for transition to school show a focus on five domains including physical and motor skills, social and emotional competencies, language development, approaches to learning, and cognitive development (National Association for the Education of Young Children [NAEYC], 1996). In practice, preschool education places a disproportionate emphasis on the cognitive aspects of school readiness while the social and emotional aspects receive less attention (Shoshani and Aviv, 2012).

An alternative prism suggests that focus on the cognitive domain and subjective well-being can be synergistic and that both are crucial in the current preschool reality. The developmental cascade model suggests that children’s seemingly distinct cognitive and emotional milestones are intertwined and progressively affect each other over time (Masten and Cicchetti, 2010). Traditional educational policies, based on the belief that focusing on children’s academic attainment is inversely related to children’s well-being and mental health, should be reconsidered. Likewise, the notion that investing more time in well-being and health leads to the neglect of academic learning and subsequently in lower achievements should also be reviewed (Bonell et al., 2013).

In addition to the synergism between the cognitive and socio-emotional domains, a focus on promoting mental health among preschoolers is important in and of itself. Early socio-emotional development provides a blueprint for subsequent mental health since it lays either a sturdy or a frail scaffold for positive or negative trajectories (Shonkoff and Phillips, 2000). Indeed, the prevalence of emotional disorders among young children is receiving worldwide attention (Côté et al., 2009). Studies report high levels of chronic mild to moderate mental health and behavior problems, particularly among young children from low socio-economic status families (Qi and Kaiser, 2003). The United States National Early Intervention Longitudinal Study identified 10–40% of infants and toddlers studied as having behavioral and emotional difficulties (United States Department of Education, 2001).

The early emergence of behavioral problems prompts the need for promoting a strong social and emotional base in preschool settings. Referral to mental health services at this age is particularly challenging, making the preschool a natural context for attending to children’s socio-emotional needs and promoting strengths, competencies and positive developmental trajectories (Hemmeter et al., 2006). In this regard, positive education can serve a dual function, providing a primary prevention platform for mental health problems that implements universal intervention practices, and facilitating subjective well-being, sense of happiness and self-actualization (Rones and Hoagwood, 2000; Shoshani and Slone, 2013; Slone and Shoshani, 2014).

### Dimensions of Well-Being of Young Children

Despite the recognized and undisputed importance of young children’s well-being, the literature is equivocal regarding the nature and elements of well-being and the paths toward its measurement and promotion. The abstract, multi-dimensional, and culturally constructed nature of the concept has led to an inconsistency in its definition in different fields (Barblett and Maloney, 2010). The classic notion of subjective well-being was operationalized as the relatively high presence of positive affect, the low presence of negative affect, and satisfaction with life (Myers and Diener, 1995). In the educational domain, well-being has been conceptualized both as an outcome and as a process that facilitates children’s advancement toward content learning and other developmental milestones. However, the study of well-being among children in the educational context is scarce compared to that of adolescents (Mashford-Scott et al., 2012).

Recently, Seligman (2011) proposed a model featuring five conditions that enable well-being among children and adults- Positive emotions, Engagement, Positive Relationships, Meaning, and Achievement that form the acronym “PERMA”- and documented the model’s applicability in educational settings. The intervention constructed and implemented in the present study was based on this model, adjusted for applicability to young children. Due to the difficulty in conceptualizing and facilitating meaning among young children (Shoshani and Russo-Netzer, 2017), the element of meaning was not included in the present intervention program.

The aspect of positive emotions in the PERMA model relates to various feelings of happiness including joy, pleasure, and fun. Several studies have confirmed a positive relation between emotional intelligence and children’s tendencies toward prosocial behavior (Eisenberg and Fabes, 1998; Luengo Kanacri et al., 2017). A positive relation between emotional understanding and mothers’ reports of children’s prosocial behavior has been shown even among young toddlers (Ensor and Hughes, 2005). Among older children aged 5, 9, and 13, comprehension of emotional expression was found to be directly associated with empathy. In turn, the positive relation between empathy and prosocial behavior has been supported, although this relation was found to weaker for girls than boys (Roberts and Strayer, 1996). School-based interventions promoting socio-emotional learning and awareness of positive emotions have demonstrated increased positive attitudes and behaviors oriented toward learning (Elias et al., 1997; Graziano et al., 2007). In addition, positive emotions such as gratitude and appreciation have been found to promote enhanced positive affect in classrooms (Froh et al., 2009).

The second element of well-being in the PERMA model is engagement which is defined as a sense of involvement and absorption in an activity, otherwise known as a state of “flow” (Csikszentmihalyi, 1991; Seligman, 2011). Studies have shown that the cultivation of children’s socio-emotional skills is associated with increased bonding to the school and adherence to norms (Elbertson et al., 2009). Additionally, classroom environments that encourage engagement have been associated with interest, attention, and curiosity during learning (Krapp, 1999).

The third aspect of the PERMA model refers to positive relationships defined as a perception of receiving support from others together with feelings of connection and security with others (Seligman, 2011). Positive and secure relationships with family, friends and peers are crucial in young children’s social-emotional development (Denham et al., 2003). Children’s ability to form positive relationships with adults enables the development of a secure basis for emotional development (Kochanska, 2001) and promotes the acquisition of social skills, self-confidence and self-esteem (Schneider et al., 2001; Gillath et al., 2005). Secure and stable relationships provide the opportunity for children to discover the effects of their behaviors on others and to gain control over the environment (Hyson, 2004).

Finally, the aspect of achievement in the PERMA model relates to a drive or ambition to accomplish personal goals (Seligman, 2011). The determination to meet challenges and maintain interest in goals over time, even when hindered with failure, have been associated with children’s life satisfaction (Peterson et al., 2007; Duckworth and Quinn, 2009). Similarly, high levels of self-control and persistence have been associated with children’s well-being (Howell, 2009).

Research has provided relatively consistent evidence that positive psychology interventions that aim to advance the PERMA factors can increase subjective well-being, prosocial behavior, and a sense of achievement and decrease mental health problems among school children (Eades, 2005; Slone and Shoshani, 2006; Boehm and Lyubomirsky, 2009; Duckworth and Quinn, 2009; Morris, 2009; Seligman et al., 2009; Williams, 2011; Slone et al., 2013; Shoshani and Steinmetz, 2014; Shoshani et al., 2016). However, findings are lacking for the potential benefits of positive psychology interventions for young children.

### The Current Positive Education Intervention – the Maytiv Preschool Program

The Maytiv preschool program was constructed by a team of psychologists in an institution of higher education in Israel. The program was administered by preschool teachers, trained and guided in the elements of the intervention. The program focused on four elements of the PERMA model with activities for enhancement of positive emotions, engagement, positive relationships and achievement.

The module for facilitating positive emotions addressed emotional expression, emotional regulation, empathy, positive thinking, and the ability to differentiate between positive and negative feelings and to express both freely. Examples of activities in this module included identification of personal sources of happiness, exercises for expressing gratitude, free expression of different feelings in movement, art, speech and facial expressions, and descriptions of memories of happy experiences. The second module dealing with engagement aimed to cultivate interest and enjoyment in activities based on encounters with personally fulfilling experiences. Examples of activities in this module consisted of playing with personally meaningful toys brought from home to the kindergarten, the opportunity to select a personally enjoyable topic or activity to the morning group meeting, identifying and using personal character strengths in the different roles adopted in the daily activities in the kindergarten. The third module aimed to facilitate positive social relationships and was based on the ability for positive communication with peers and adults, support for prosocial behavior and cooperation, and encouragement of acts of kindness and empathy. Illustrative activities in this module included games that demanded peer cooperation, exercises in playing with friends in different situations, encouragement of offering positive responses to other children, simulated conflict resolution and consideration for a friend’s feelings. The fourth module of achievement addressed identification and pursuit of goals and personally significant objectives. Activities in this module included games that emphasized perseverance in challenging situations, games that provided sense of efficacy and support for willingness to continue trying despite failure, and selection of and working on personal projects such as producing a book of drawings or exploring interesting topic.

The purpose of the present study was to examine the efficacy of the Maytiv Preschool program in promoting mental health, subjective well-being and adaptive preschool functioning as compared to a matched waiting-list control group in a pre-test to post-test repeated measures design.

The central hypothesis of the study predicted that participants in the intervention group would exhibit a greater pre- to post-intervention increase in their subjective well-being (higher positive emotions and life satisfaction and lower negative emotions), mental health (lower emotional and behavioral problems), and adaptive preschool functioning (self-regulation, empathy, pro-sociality, and positive approaches to learning) than participants the control group. In addition, the exploratory question examined whether efficacy of the intervention differed across socio-demographic background characteristics, such as the child’s age, gender, and family socio-economic status.

## Materials and Methods

### Participants

Participants were 315 preschool children (153 girls, 162 boys) aged 3–6.5 (*M* = 4.53, *SD* = 0.93) from 12 demographically similar preschool classrooms in a central city in northern Israel. In Israel, kindergarten is part of the preschool system.

State kindergartens in Israel cater for children from 3 to 6 years old, in three age groups: ages 3–4, 4–5, and 5–6.

Assignment to study conditions followed a two-step process. First, 42 preschools were selected from 64 preschool classrooms in the same geographic area. Exclusion criteria for preschools were ultra-orthodox religious preschools, special education, a small number of children, or demographically incomparable preschools. Eighteen preschool teachers expressed interest in participating in the program and agreed to a random selection process.

In the second stage, six of the interested preschools were randomly selected for the intervention and six were allocated to a wait list control group. All 352 children in the 12 preschools were eligible to participate in the study. All parents complied with informed consent requirements, except for five parents who refused consent for their children participating in the assessments in the study. A total of 32 children did not complete the study; 18 due to absence and 14 due to refusal to participate in the second measurement. Thus, the intervention groups comprised 160 and the control 155 children who completed both the pretest and posttest measures. Both groups were approximately evenly divided by gender (48.5% boys, 51.5% girls). The study population consisted of Jewish children, 98% of them were Israeli born. A total of 189 parents also agreed to participate in the study and completed questionnaires about their children, of which 86.7% were the mothers. The age range of the parents at the beginning of the study was 24–52 years (*M* = 36.29, *SD* = 4.70), 1.5% were single, 92% were married and 6.5% were divorced. Sixteen parents dropped out of the study at the second measurement.

### Measures

#### Measures for Children

*The Shortened Positive and Negative Affect Scale for Children (PANAS-C)* (Ebesutani et al., 2012) is the brief version of the PANAS-C (Laurent et al., 1999), a standardized measure that assesses levels of positive and negative emotions experienced over the previous few days. The measure comprises 10 adjectives that relate to five positive emotions (cheerful, happy, joyful, proud, lively) and five negative emotions (mad, miserable, afraid, sad, scared), rated on a 5-point Likert scale. The scale has not been validated for children below age 7 due to difficulties in administering paper-and-pencil questionnaires to young children. Therefore, in this study, we modified the scale in several ways. First, we read aloud the items to children individually. Second, we simplified some of the complex wording (positive emotions – joyful, happy, glad, excited, and proud; negative emotions – angry, unhappy, afraid, scared, and sad). We also changed the response scale from the original 5-point Likert scale to a 3-point pictorial Likert scale. The scale showed a series of three boxes ranging from empty to full and we asked the participants to indicate whether they felt the emotion none of the time (empty box), sometimes (half full box), or all of the time (full box). In this study, alpha coefficients were 0.89 and 0.86 at time 1 for the positive and negative affect subscales respectively.

*Brief Multidimensional Students’ Life Satisfaction Scale* (BMSLSS) (Seligson et al., 2003) is a self-report five-item measure for children and adolescents that assesses life satisfaction in different domains. On a 7-point scale ranging from 1 (terrible) to 7 (delighted). Each item assesses life satisfaction in one of five areas (e.g., “I would describe my satisfaction with my family life/friendships/school experience/myself/where I live as...”). The scale was modified for suitability for young children by changing the item to reflect the children’s rating of satisfaction with their families, friends, preschool, home, and self-experiences depicted by little shadow figures representing the domain. The child-adapted instructions were “How happy are you with your ….” The rating scale was adapted from the numbers 1 – 7 to a 5-point scale depicted by a series of smiley faces ranging from a large green smiling face, a smaller green smiling face, a neutral half green, half red face, a larger red sad face, and a large red sad face. The present study yielded α = 0.80 on the pre-test measurement.

*Affective Situations Test for Empathy (FASTE)* (Feshbach and Roe, 1968) is measure designed to assess empathy and consists of eight narratives, accompanied by three slides each, that describe hypothetical daily life situations that arouse emotion. The instrument presents narratives and a series of slides depicting a young child in four different affective situations, with two sequences for each situation – happiness, sadness, fear, and anger. The instrument contains alternative depictions according to gender. The contents for each of the four affective situations were: (1) Happiness – birthday party, winning a television contest; (2) Sadness – a lost dog, social rejection; (3) Fear – lost child, frightening dog; (4) Anger – a child snatching away a toy, false accusation. Children report their feelings concerning each situation and reports were recorded verbatim. Responses are scored with two points for a match between the situation observed and the child’s response, one point for a consistent negative or positive response that was inaccurate, and no points for an inconsistent or irrelevant emotion. A total empathy score for each child was assessed as the sum of empathic responses to all of the affective situations.

*The Head-to-Toes task (HTKS)* (McClelland and Cameron, 2012) was used to evaluate children’s behavioral self-regulation. The task requires cognitive flexibility, and inhibitory control and working memory. Children are required to first follow one of two commands for action and then are requested to respond with a conflicting, non-automatic action. For example, if the experimenter says, “Touch your head,” the correct response would be to touch one’s toes. Task complexity is increased with additional conflicting actions. After a practice session, children perform the task according to instructions from the experimenter. The measure is scored with two points for a correct action, one point for a self-correcting actions and no points for an incorrect action, such that the scores for the 20 item scale ranged from 0 to 40.

#### Measures for Parents

*The Parent Version of the 10-Item Positive and Negative Affect Schedule for Children (PANAS-C-P)* (Ebesutani et al., 2012) is a 10-item scale presented to the parents who assess their child’s positive and negative affect. Parents rate the extent to which their child had displayed each mood in the previous few days on a five-point Likert scale ranging from 1-very slightly- to 5- extremely. Research has supported convergent and discriminant validity of the scale with measures of childhood anxiety and mood disorders and high internal consistency (Ebesutani et al., 2012). In this study, alpha coefficients were 0.93 and 0.91 at time 1 for the positive and negative affect subscales respectively.

*Strengths and Difficulties Questionnaire (SDQ)* (Goodman et al., 1998) is a 25-item self-report instrument that assesses mental health disorder. The questionnaire yields a total difficulties score and five scales consisting of five items each – Emotional symptoms (e.g., I am often unhappy, depressed or tearful), Conduct problems (e.g., I take things that are not mine from home, school or elsewhere), Hyperactivity scale (e.g., I am constantly fidgeting or squirming), Peer problems (e.g., I am usually on my own) and a Prosocial scale (e.g., I often volunteer to help others). In this study, ratings on the four difficulties scales were summed to produce a total difficulties score. The fifth scale, the prosocial scale, was calculated and constituted part of the adaptive preschool functioning outcome variable. This study used the Hebrew version of the SDQ as translated by the Israeli Ministry of Health. The instrument demonstrates good criterion validity for both community and clinic samples and high cross-informant correlations between self-report to parent- and teacher-reports (Goodman et al., 1998). The Hebrew version has yielded good internal consistency (α = 0.51–0.72) and good construct, concurrent, and discriminant validity (Mansbach-Kleinfeld et al., 2010). In this study, Cronbach’s alphas were good for the total difficulties score (0.79), and for the four problem scales (0.72–0.81).

#### Measures for Preschool Teachers

*Approaches to Learning Scale* (Zill and West, 2001) assesses approaches to learning behaviors in preschool children. The seven item teacher-report scale evaluates children’s learning behaviors in seven areas: attentiveness, eagerness to learn, task persistence, learning independence, organization, flexibility, and ability to follow classroom rules. Scores range from 1 to 4, with higher scores indicating more frequent displays of positive learning behaviors. Scores on this scale have been correlated with reading, mathematics, and science scores in kindergarten and first grade and the scale shows good criterion validity (Entwisle and Alexander, 1998). In this study, the Alpha Coefficient was 0.78 on the pre-test.

### Intervention and Control Conditions

#### Intervention Program

The intervention program included two parallel phases. One phase consisted of a preschool teachers’ training workshop led by a clinical psychologist trained in group dynamics and in positive psychology. The second phase consisted of the parallel administration by preschool teachers of an age-appropriate program to the children in the classroom.

In the first phase, consisting of a positive psychology training workshop, preschool teachers took part in a 34 academic hour workshop that included two 90-min introductory sessions and 15 bi-weekly 90-min lessons from September 2016 to June 2017. The teacher workshops were conducted in the afternoon in the local teachers’ training center. The workshops provided teachers with an introduction to positive psychology, explanations of the content, provision of tools that could be personally useful, and provision of materials to be imparted to the children. Preschool teachers were encouraged to engage actively in the learning process in order to increase assimilation of the material. This engagement took the form of personal sharing, shared exercises during the workshop and homework exercises. They were also provided with a textbook consisting of techniques for teaching the curriculum and implementing the lessons in their preschool classes. Although difficult to monitor, preschool teachers were requested to avoid sharing this material outside of the workshops. All participating preschool teachers gave informed consent and none refused participation even when offered the opportunity to do so.

The children’s program was delivered in the classroom during the preschool day. In order to ensure standardization of the intervention, preschool teachers were equipped with a textbook containing curriculum materials and plans of all the lessons. The curriculum consisted of four content modules, each of which consisted of two subject units. The basic units were: positive emotions (expression and management of emotions and gratitude), engagement (love of learning and character strengths), achievement (focusing and persistence), and positive relationships (positive relationships and empathy). The textbook contained theoretical material concerning each module and a rich and varied set of activities for each session containing discussions, stories, songs, games, and activities that could be combined into the regular preschool daily activities such as during artwork time and free play. Each unit contained 20 activities that were delivered over a period of 1 month, 5 activities per week, such that the program began in October 2016 and continued for 32 weeks.

##### Implementation fidelity

After each monthly module, preschool teachers were requested to complete a report that evaluated the extent to which the teachers adhered to the lesson plan. Teachers reported whether they had managed to deliver all activities on a polar question for each component. Analysis of the fidelity reports revealed that teachers implemented on average 18 of the 20 activities in each unit (*M* = 18.27, *SD* = 0.74). All the teachers reported enthusiastic participation by the children and excellent cooperation.

### Control Group

The control group was a no-treatment waiting list passive control group. The control classrooms did not have any positive psychology lessons nor did they participate in any other social-emotional curriculum during the implementation of the intervention, but rather continued with the regular preschool curriculum and activities.

The control group participated in the evaluation study at the two measurement time points and was placed on the waiting list for the Maytiv preschool program after completion of the study.

### Procedure

Data were collected in two waves, time 1 in September 2016 at the beginning of the Israeli school year, and time 2 in June 2017, at the end of the school year. After receiving authorization for the study from the Israeli Ministry of Education’s ethics committee and the Herzliya Interdisciplinary Center Academic Ethics Committee, research assistants contacted the preschool teachers and parents and received written informed consent. In order to ensure that consent was informed, teachers and parents were supplied with information about the research, intervention, control and randomization process. Parents signed consent for their children to participate in the intervention and in the research and to complete questionnaires about their children themselves. Parents received a $20 gift voucher for their participation in each time wave. A total of 89 parents from the intervention group and 64 parents from the control group consented to participation and completed questionnaires at both time points. An additional 18 parents completed questionnaires at time 1 but failed to complete the study. Teacher and Parent questionnaires were completed electronically using the Qualitrics platform

In order to ensure confidentiality, participants were assigned an identification number and were re-identified only for follow-up purposes. Additionally, provisions for debriefing or counseling were available to any participant who felt negatively impacted from participation in the program or study. None of the teachers, parents or children sought counseling services from the Maytiv team during or following the program.

Each child was taken individually by an experimenter from the classroom to the experimental room, a quiet and pleasant private room in the preschool. Children were informed that they were going to see pictures, perform tasks, and hear stories about children their own age. Tasks and questionnaires were presented on a laptop screen and were administered in a counterbalance order. The duration of the data collection procedure was approximately 20 min.

### Data Analysis

All statistical analyses were performed with SPSS software (version 24.0). Baseline differences in demographic characteristics between the groups were analyzed with χ2 analysis for discrete variables and independent sample *t*-tests for continuous variables. Changes in the outcome variables were examined with repeated measures Analyses of Variance (ANOVAs), using type of intervention with two levels (intervention and control group) and time with two levels (pre- and post-intervention), as factors. The within-subjects factors were positive and negative affect (child and parent report), life satisfaction, self-regulation, empathy, mental health difficulties (the total score of the SDQ), prosocial behavior, and approaches to learning. The Bonferroni correction was used to account for multiple comparisons (*p* < 0.005). Cohen’s *d* was used to estimate the magnitude of intervention effect sizes at post-intervention relative to baseline status. Missing data accounted for less than 2% for all the study variables and a multiple imputation method was utilized to impute missing values.

## Results

### Description of Sample at Baseline

**Table 1** summarizes the demographic information and baseline characteristics of the participants in each group. Baseline comparisons revealed no significant pre-intervention differences between the intervention and the control group on the demographic or baseline measures.

**Table 1:** Sample characteristics at baseline.

	Control group (*n* = 155)	Intervention group (*n* = 160)	Statistic	*P*
Gender				
Girls, *n* (%)	72 (46.4%)	81 (50.6%)	χ^2^= 1.80	0.20
Age (years); *Mean* (SD)	4.45 (0.84)	4.58 (0.83)	*t* = 1.38	0.16
Socioeconomic status (PR)				
High middle class *n* (%)	17 (20.2%)	15 (16.8%)	χ^2^= 1.18	0.55
Middle class	50 (59.6%)	60 (67.4%)		
Low middle class	17 (20.2%)	14 (15.8%)		
Preschool levels				
Ages 3–4, *n* (%)	50 (32.3%)	49 (30.6%)	χ^2^= 0.11	0.95
Ages 4–5, *n* (%)	54 (34.8%)	58 (36.3%)		
Kindergarten, ages 5–6.5, *n* (%)	51 (32.9%)	53 (33.1%)		
Positive emotions- CR	12.59 (2.33)	12.19 (2.35)	*t* = 1.51	0.13
Negative emotions – CR	9.48 (2.90)	9.73 (2.90)	*t* = 0.76	0.44
Empathy- CR	9.08 (5.23)	8.91 (5.25)	*t* = 0.29	0.77
Self-regulation- CR	23.38 (13.29)	20.12 (15.97)	*t* = 1.96	0.06
Life satisfaction- CR	3.88 (0.81)	3.82 (0.74)	*t* = 0.69	0.49
Positive emotions- PR	19.44 (4.62)	19.39 (3.91)	*t* = 0.10	0.91
Negative emotions – PR	11.37 (3.41)	11.63 (3.52)	*t* = 0.67	0.51
SDQ- total difficulties- PR	24.35 (4.01)	24.53 (4.37)	*t* = 0.38	0.70
Prosocial- PR	12.06 (1.91)	12.14 (1.87)	*t* = 0.38	0.71
Approaches to Learning-TR	3.20 (0.34)	3.12 (0.43)	*t* = 1.82	0.07

*CR, child report; PR, parent report; TR, teacher report; SDQ, strengths and difficulties questionnaire; *N* for parents of intervention group is 89 and of control group is 84.*

Correlations for the measured variables in the study at baseline are presented in **Table 2**. Children’s age was positively related to positive affect, life satisfaction, empathy, self-regulation, positive approach to learning, and lower mental health problems. Boys were reported to have more behavioral problems and less empathy, and positive and negative emotions than girls. Children’s positive emotions were positively related to life satisfaction, prosocial behavior, and less emotional problems. Life satisfaction was also related to the child’s empathy and self-regulation.

**Table 2:** Bivariate correlations between baseline variables.

		2	3	4	5	6	7	8	9	10	11	12	13	14	15	16
1	Age	0.08	0.11	0.01	0.22***	0.33***	0.41***	0.01	–0.18*	–0.12	–0.31***	0.04	–0.25**	–0.17*	–0.23**	0.29***
2	Gender (boys)	–	–0.03	–0.02	–0.09	–0.12*	–0.07	–0.17*	–0.18*	0.10	0.17*	–0.15	0.00	0.01	0.03	–0.17*
3	Positive emotions- CR		–	–0.21***	0.25***	0.01	–0.03	0.28***	–0.29***	–0.34***	–0.10	0.08	–0.22**	0.23**	–0.22**	0.17*
4	Negative emotions – CR			–	–0.16**	0.26***	–0.18***	–0.24**	0.20*	0.23**	0.07	0.10	0.12	–0.17*	0.19*	–0.14
5	Life satisfaction- CR				–	0.16**	0.24***	0.19*	–0.12	–0.12	–0.10	0.22**	–0.02	0.19*	–0.10	0.01
6	Empathy- CR					–	0.30***	–0.13	–0.06	–0.22**	–0.14	0.12	–0.12	0.12	–0.19*	–0.09
7	Self-regulation- CR						–	0.07	–0.09	–0.05	–0.22**	–0.29***	–0.25**	0.04	–0.09	0.15
8	Positive emotions- PR							–	–0.28***	–0.29***	–0.25**	–0.02	–0.25**	0.33***	–0.30***	0.37***
9	Negative emotions – PR								–	0.54***	0.35***	0.13	0.32***	–0.48***	0.50***	–0.23**
10	Emotional problems- PR									–	0.54***	0.22**	0.26**	–0.52***	0.79***	–0.32***
11	Conduct problems- PR										–	0.11	0.51***	–0.67***	0.82***	–0.30***
12	Hyperactivity- PR											–	0.09	–0.11	0.47***	0.01
13	Peer problems- PR												–	–0.54***	0.63***	–0.28***
14	Prosocial- PR													–	–0.68***	0.35***
15	Total difficulties- PR														–	–0.34***
16	Approaches to Learning-TR															–

*CR, child report; PR, parent report; TR, teacher report.*

### Intervention Effects

Comparisons of mean changes between intervention and control groups are presented in **Table 3**. None of the study variables showed significant kurtosis or skewness that would necessitate transformation.

**Table 3:** The intervention and control groups’ scores on the study measures before and after the intervention.

	Control (*n* = 155)					Intervention (*n* = 160)				
	T1		T2			T1		T2		
	Mean	*SD*	Mean	*SD*	*d*	Mean	*SD*	Mean	*SD*	*d*
Positive emotions- CR	12.59	2.33	12.64	2.21	0.02	12.19	2.35	13.03	2.11	0.38
Negative emotions – CR	9.48	2.90	10.27	3.08	0.26	9.73	2.90	9.89	2.96	0.05
Empathy- CR	9.08	5.23	9.65	4.55	0.12	8.91	5.25	10.92	6.55	0.34
Self-regulation- CR	23.38	13.29	25.62	12.31	0.18	20.12	15.97	23.12	15.30	0.19
Life satisfaction- CR	3.88	0.81	3.95	0.80	0.09	3.82	0.74	4.28	0.63	0.67
Positive emotions- PR	19.44	4.62	19.31	4.09	0.03	19.39	3.91	22.11	2.72	0.81
Negative emotions – PR	11.37	3.41	11.33	3.04	0.01	11.63	3.52	11.29	3.29	0.10
Hyperactivity- PR	5.74	1.95	5.22	0.90	0.34	5.92	1.95	5.21	0.89	0.47
Peer problems- PR	6.04	1.49	5.36	0.76	0.58	5.76	1.13	5.21	0.70	0.59
Emotional problems- PR	7.00	1.53	6.50	1.18	0.37	7.11	1.91	6.64	1.22	0.29
Conduct problems- PR	5.57	1.47	6.33	0.99	0.61	5.74	1.50	6.43	1.33	0.49
Total difficulties- PR	24.35	4.01	23.42	2.63	0.28	24.53	4.37	23.49	2.96	0.28
Prosocial- PR	12.06	1.91	11.50	2.28	0.27	12.14	1.87	13.29	1.61	0.66
Approaches to Learning-TR	3.20	0.34	3.07	0.38	0.36	3.12	0.43	3.39	0.45	0.62

*CR, child report; PR, parent report; TR, teacher report; Cohen’s *d* effect sizes are considered as large ≥ 0.8; medium 0.5–0.7; small ≤ 0.4.*

We first conducted exploratory analyses to examine potential covariates of subjective well-being and mental health change over the year, including age, grade level, gender, family status, socio-economic status, and the baseline variables. Age was the only variable that was significantly correlated with one of the outcome variables (the conduct problems modification), and so was included as a covariate in the relevant analysis. The effects of the intervention were examined using repeated measures analysis of variance, with time as a within-subjects factor and experimental condition as a between-subjects factor.

Analyses of the self-report outcomes showed significant interaction effects between intervention and time (pre- and post-intervention) on children’s positive emotions, *F(*1,313) = 10.93, *p* = 0.001, η^2^ = 0.04, life satisfaction, *F(*1,313) = 9.68, *p* = 0.002, η^2^ = 0.03, and empathy, *F(*1,313) = 8.65, *p* = 0.004, η^2^ = 0.03, but not on negative emotions, *F(*1,313) = 1.83, *p* = 0.18, η^2^ = 0.007. *Post hoc t*-test comparisons revealed that children in the intervention group showed significant pre- to post-intervention increases in positive emotions (*M* = 0.84, *p* < 0.001, *Cohen’s d* = 0.38), life satisfaction (*M* = 0.46, *p* < 0.001, *Cohen’s d* = 0.67) and empathy (*M* = 2.01, *p* = 0.003, *Cohen’s d* = 0.34), with no significant changes in these measures in the control group. There was no significant interaction effect between intervention and time on negative emotions. For self-regulation, there was a significant positive effect of time, *F*(1,313) = 9.82, *p* = 0.002, η^2^ = 0.03, but no significant interaction of time × intervention, *F*(1,313) = 0.21, *p* = 0.65, *η*2 = 0.001.

Analyses of the parent-report data showed a similar pattern for children’s positive emotions of time x intervention significant effect, *F(*1,171) = 9.11, *p* = 0.004, η^2^ = 0.02, with significant increases in the intervention group (*M* = 2.72, *p* < 0.001, *Cohen’s d* = 0.81), and no significant changes in the control group. For prosocial behavior, we also found a significant increase in the intervention group, *F(*1,171) = 7.29, *p* = 0.004, η^2^ = 0.03, (*M* = 1.15, *p* < 0.001, *Cohen’s d* = 0.66), while there was no significant change in the control group. There were no significant differences between the intervention and the control groups in changes in negative emotions, *F*(1,171) = 1.78, *p* = 0.19, η^2^ = 0.05, and in total mental health difficulties, *F*(1,171) = 1.63, *p* = 0.18, η^2^ = 0.04. However, there was a main effect of time on total mental health difficulties, *F*(1,171) = 7.52, *p* = 0.002, η^2^ = 0.04, with a significant decrease in difficulties from pre- to post-intervention in both the intervention and control groups. The time × age interaction effect was not significant (*p* = 0.13). A beneficial effect of the intervention was also found on the teachers’ reports on children’s approaches to learning, *F*(1,171) = 10.15, *p* = 0.003, η^2^ = 0.21, with significant increase over the year in positive learning behaviors and engagement in the intervention group (*M* = 0.31, *p* < 0.001, *Cohen’s d* = 0.62), but with no significant changes in the control. Cohen’s *d* effect sizes for the magnitude of the significant changes in the intervention group were in the small to large range (0.34–0.81) according to Cohen (1988).

## Discussion

This research represents the first study to advance and examine the efficacy of a positive education program for preschool children. Despite the recognized value of positive education, its importance at the preschool level has lagged behind in this field. Educators and parents place great importance on children’s happiness and well-being, yet the field of developmental positive psychology has not gained sufficient attention at the research and practical levels. The focus of the study and subsequent findings highlight the importance of integrating positive psychology contents into children’s daily activities in the preschool.

The central hypothesis of this study contained three dimensions of functioning – subjective well-being, mental health and preschool functioning – in order to enable a broad evaluation of the effects of the intervention. The first part of the hypothesis predicted pre- to post-intervention increases in well-being in the intervention, as compared to the control group. Findings showed a significant increase in the intervention but not in the control group in children’s positive emotions and no change in negative emotions as reported by both parents and children themselves. In addition, findings indicated significant increases in children’s self-report of life satisfaction, which is not a trivial finding given that the period of early childhood is usually characterized by many natural opportunities for positive emotions in play, games and enjoyment. Results suggest that positive and negative emotions are not always interdependent. The findings here could reflect the dominance of promoting positive emotions in the intervention program or the greater ease with which programs can facilitate positive emotions rather than reduce negative emotions. This assumption could explain the lack of change in self-regulation, although this was also a focus of the program. It is possible that management of negative emotions demands more time than was available in this particular program, especially among young children whose regulatory abilities are relatively undeveloped.

The second part of the hypothesis predicted a greater pre- to post-intervention decrease in children’s mental health difficulties in the intervention as compared to the control group. This part of the hypothesis was not confirmed. The lack of significant findings for changing mental health difficulties fits with the above argument that this preschool intervention program was more effective in promoting strengths than in reducing difficulties. This accords with recent models that conceptualize mental health as being comprised of the two distinct factors of mental illness and difficulties and subjective well-being (Greenspoon and Saklofske, 2001). The presence of high levels of subjective well-being does not automatically imply the absence of mental health difficulties (Sin and Lyubomirsky, 2009). Possibly, interventions that aim to address both dimensions necessitate more therapeutic elements and much more involvement of the family and other caretakers. Nonetheless, the value of successfully enhancing positive emotions and life satisfaction should not be underestimated, given the importance of these aspects in children’s mental health.

The third part of the hypothesis predicted a greater pre- to post-intervention increase in children’s adaptive preschool functioning than the control group participants. Our findings indicated significant increases in children’s empathy, prosocial behavior, and positive approaches to learning in the intervention group with no significant changes over time in these measures in the control group. These results indicate that, beyond the personal emotional changes that occurred, the effects of the intervention program extended to the interpersonal sphere. The program included an emphasis on identification of feelings and interpersonal discussions about feelings. This component possibly increased the ability to understand the other’s feelings and opened opportunities for giving and for prosocial behavior.

Another extended effect related to approaches to learning and general preschool functioning. Preschool functioning was measured as enthusiasm for learning, attention, persistence, autonomy, flexibility, organization, and adherence to rules, all of which form the base for a sense of achievement and acquisition of personal goals. These aspects lay the foundation for learning skills and engagement with learning, which are important qualities that will influence subsequent academic success (Seligman, 2011).

Analyses also revealed some age and gender baseline differences in the study variables. For example, children’s age was positively related to higher subjective well-being and lower mental health problems at the beginning of the study. In addition, boys had more behavioral problems and this corresponds to the higher levels of externalizing symptoms attributed to preschool-age boys (Zahn-Waxler, 2000), and less positive and negative emotions than girls. The lack of studies on subjective well-being in preschool children makes it difficult to interpret these gender and age variances and suggests that much more research needs to be conducted in this area of positive psychology in early childhood. However, despite these differences, there were no significant interactions between the demographic variables examined in this study and the intervention. This finding possibly implies that the program is relevant and meets the needs of children of different genders, ages, and socioeconomic backgrounds.

### Limitations

A study of this type involves multiple challenges including development of appropriate positive education contents adjusted for this young age group, logistic difficulties in applying the program, and utilization of suitable instruments and strategies for measurement of positive psychology-based variables. This study met these challenges by using multi-informant agents and innovative program activities and assessment tools.

However, there are several limitations that must be recognized in this study. This study did not include an evaluation of the effects of the program effects for teachers who had participated in the positive psychology training. Possibly teachers’ personal connection to the program content could have effected the intervention by exceeding merely adhering to the guidelines and ensuring fidelity.

The positive effects may also be explained by a Hawthorne effect, which refers to the possibility the participants in the intervention group showed positive changes as a result of being treated differently and receiving more attention (Adair, 1984). The absence of an active control group complicates determination of the influence of additional components, beyond the special attention and close relationships with the teachers, that may have been responsible for the effects. In addition, the intervention could be vulnerable to the issue of demand characteristics (Nichols and Maner, 2008), especially given the emphasis on positive factors that were included in the self-report measures.

The present study used a repeated measures design at only two time points with no delayed post-test follow-up. In addition, in view of the potential for later developing effects, as posited in positive psychology (Seligman et al., 2009) there is a necessity to trace the long-term effects of the seeds sewn in early childhood positive education interventions with longitudinal studies.

### Implications

Kindergarten teachers are in a unique position to apply interventions that can strengthen children’s well-being at a young age. At the practical level, the intervention in this study equipped preschool teachers and children with practices and materials aimed at promoting well-being. Although there are multiple pathways to promote well-being (Seligman, 2011), the Maytiv program was constructed as an integrative intervention that applies to children with varying needs and from diverse backgrounds.

This research is unique in being the first efficacy study of a positive psychology program for preschool children worldwide. Many preschool interventions have been developed and studied. However, the present program contains many elements proven as valuable to well-being in the youth and adult literature, but not emphasized in preschool education. Gratitude, virtues, character strengths, perseverance, kindness and positive social relationships are valuable and foundational qualities for well-being from the earliest ages and across all ages.

## Ethics Statement

This study was carried out in accordance with the recommendations of the Herzliya Interdisciplinary Center Academic Ethics Committee and the Israeli Ministry of Education with informed consent from all participants. Parents gave written informed consent in accordance with the Declaration of Helsinki. The protocol was approved by the Israeli Ministry of Education’s ethics committee and the Herzliya Interdisciplinary Center Academic Ethics Committee.

## Author Contributions

All authors listed have made a substantial, direct and intellectual contribution to the work, and approved it for publication.

## Conflict of Interest Statement

The authors declare that the research was conducted in the absence of any commercial or financial relationships that could be construed as a potential conflict of interest.
